# Patients’ Needs and Requirements for eHealth Pain Management Interventions: Qualitative Study

**DOI:** 10.2196/13205

**Published:** 2019-04-01

**Authors:** Ingrid Konstanse Ledel Solem, Cecilie Varsi, Hilde Eide, Olöf Birna Kristjansdottir, Jelena Mirkovic, Elin Børøsund, Mette Haaland-Øverby, Karina Heldal, Karlein MG Schreurs, Lori B Waxenberg, Karen Elizabeth Weiss, Eleshia J Morrison, Lise Solberg Nes

**Affiliations:** 1 Center for Shared Decision Making and Collaborative Care Research Division of Medicine Oslo University Hospital Oslo Norway; 2 Institute of Clinical Medicine Faculty of Medicine University of Oslo Oslo Norway; 3 Science Centre Health and Technology University of South-Eastern Norway Drammen Norway; 4 Norwegian National Advisory Unit on Learning and Mastery in Health Oslo University Hospital Oslo Norway; 5 Centre for eHealth and Wellbeing Research University of Twente Enschede Netherlands; 6 Department of Clinical and Health Psychology University of Florida Florida, FL United States; 7 Department of Anesthesiology and Pain Medicine University of Washington School of Medicine Washington, WA United States; 8 Mayo Clinic Pain Rehabilitation Center Rochester, MN United States; 9 Department of Psychiatry & Psychology Mayo Clinic Rochester, MN United States

**Keywords:** chronic pain, eHealth, self-management, qualitative methods, intervention development, user-centered design

## Abstract

**Background:**

A growing body of evidence supports the potential effectiveness of electronic health (eHealth) interventions in managing chronic pain. However, research on the needs and preferences of patients with chronic pain in relation to eHealth interventions is scarce. Eliciting user input in the development of eHealth interventions may be a crucial step toward developing meaningful interventions for patients for potentially improving treatment outcomes.

**Objective:**

This study aimed to explore the experiences of patients with chronic pain with regard to information and communication technology, understand how an eHealth intervention can support the everyday needs and challenges of patients with chronic pain, and identify possible facilitators and barriers for patients’ use of an eHealth pain management intervention.

**Methods:**

Twenty patients living with chronic pain and five spouses participated in individual interviews. Semistructured interview guides were used to explore participants’ needs, experiences, and challenges in daily life as well as their information and communication technology experiences and preferences for eHealth support interventions. Spouses were recruited and interviewed to gain additional insight into the patients’ needs. The study used qualitative thematic analysis.

**Results:**

The participants were generally experienced technology users and reported using apps regularly. They were mainly in favor of using an eHealth self-management intervention for chronic pain and considered it a potentially acceptable way of gathering knowledge and support for pain management. The participants expressed the need for obtaining more information and knowledge, establishing a better balance in everyday life, and receiving support for improving communication and social participation. They provided suggestions for the eHealth intervention content and functionality to address these needs. Accessibility, personalization, and usability were emphasized as important elements for an eHealth support tool. The participants described an ideal eHealth intervention as one that could be used for support and distraction from pain, at any time or in any situation, regardless of varying pain intensity and concentration capacity.

**Conclusions:**

This study provides insight into user preferences for eHealth interventions aiming to address self-management for chronic pain. Participants highlighted important factors to be considered when designing and developing eHealth interventions for self-management of chronic pain, illustrating the importance and benefit of including users in the development of eHealth interventions.

**Trial Registration:**

ClinicalTrials.gov NCT03705104; https://clinicaltrials.gov/ct2/show/NCT03705104.

## Introduction

Chronic pain conditions are common (affecting 25%-30% of the population) and difficult to cure, with a significant impact on the persons affected and on society in terms of economical, psychological, and social issues [1-3]. Like all long-term conditions, chronic pain requires day-to-day self-management by those affected. This includes managing the emotional and medical consequences of the chronic condition; self-regulatory efforts; and changing, maintaining, and creating new sets of behaviors to improve coping mechanisms [3-5]. Supporting patients and caregivers in the self-management process is an important step toward better health care services and have shown to have a positive impact on patient outcomes, including decreased pain interference and pain intensity and improved self-efficacy [6-9].

A growing body of evidence supports the potential efficacy of electronic health (eHealth) interventions contributing to self-management of chronic pain [10-16], which refer to interventions using information and communication technology (ICT) such as apps, websites, or remotely delivered interventions/telehealth or telecare in the delivery of health care services [17]. eHealth interventions have the potential to make health care services more available to patients, allowing patients to access services and help from their own home. In addition, eHealth interventions may introduce more cost-effective treatment options, reducing the need for travel and direct health care personnel involvement [13,18]. Such interventions also have the potential to enhance treatment durability, as patients can receive support and reinforcement of skills during and after treatment [13].

Patients with chronic pain have also shown interest in eHealth interventions [19]. For some, such interventions could even be the preferred option, as they are easily accessible, possibly perceived as neutral and nonjudgmental, and allow patients to continue treatment and support at their own pace [20]. There is, however, a gap between the commercial and scientific aspects of eHealth tools. Available apps for people with chronic pain commonly focus on physical health and include a functionality supporting monitoring/tracking, assessment, feedback, and information/education [21]. Surprisingly, a few existing apps appear to be based on theoretical and evidence-based rationale, and a few appear to be developed or evaluated using scientific methods [21-23]. In addition, few eHealth interventions are developed by, or in collaboration with, health care professionals [12,23,24].

The success of eHealth interventions depends on technology and content but, perhaps, just as much on patients’ acceptance of and adherence to the intervention [25]. Involving patients in the process appears essential in the development of effective eHealth interventions. However, several studies have pointed out the lack of user involvement in the development of such interventions [23,25-27]. Keogh and colleagues [26] described eHealth as a promising area for pain management but emphasized the need to maintain patient focus and recommended using user-centered designs as the starting point, involving patients (called users) in the entire development process [26]. The authors also stated that eHealth interventions, even when claiming to be therapeutic, are often developed in response to a technological innovation, rather than user needs [26]. Other studies have pointed out that including users in the design of eHealth interventions would allow for tailoring of individual preferences [28], and personalization and tailoring in such interventions may improve impact [20,23]. Despite these recommendations from existing research, users are rarely involved early on in eHealth development processes [25]. A recent study addressed this issue by combining a review of the literature with a focus group study including patients with chronic pain, their caregivers, and their health care providers. This resulted in a suggestion of elements needed for an eHealth intervention for people with chronic pain, including enriched information environment, automated tailored feedback observations of individual progress, automated follow-up messages, communicative function (eg, advisor or peer-support access), and use of supplementary modes (eg, access to and use of a variety of materials) [29].

The aims of this study were (1) to advance knowledge regarding the needs and requirements for eHealth pain management interventions to explore experiences of patients with chronic pain with regard to information and communication technology (ICT) in order to explore how an eHealth intervention can support the everyday needs and challenges of patients with chronic pain and (2) to identify possible facilitators and barriers for patients’ use of an eHealth pain management intervention. This study is the first step in a larger project where the aim is to design, develop, and test a user-centered eHealth intervention for adults with chronic pain based on cognitive behavioral therapy (CBT; trial registration: NCT03705104).

## Methods

### Study Design

This study used a qualitative design involving individual interviews with patients with chronic pain and their spouses [30], to explore patients’ needs and preferences for designing and developing eHealth interventions. In the study, spouses were included to gain additional insight into the perceived needs of patients, from the spouses’ perspective. Identifying and exploring patients’ needs, experiences (in everyday life as well as in relation to technology), and preferences are early steps in a user-centered development process [31,32].

### Recruitment

To be eligible for study participation, participants had to be 18 years or older, have experienced chronic pain for 3 months or more, and be able to communicate in Norwegian. Inclusion criteria for spouses were that they were married or cohabitating with one of the participating patients. Recruitment was conducted by collaborative health care providers at four collaborative institutions. Persons who met the inclusion criteria were invited to participate in individual interviews by health care providers at local patient education centers, pain clinics, physical therapy institutes, and psychology practices. Potential participants first received information about the study through hand-out pamphlets and from their health care providers. If the potential participants were interested, they were contacted by a researcher for more information. Of the people who had agreed to be contacted by the research team, none declined participation. Spouses were contacted after obtaining consent from the patients, only if the participating patient agreed that their spouse could be contacted. Participants (patients and spouses) were offered a gift certificate (value of approximately US $30) as compensation for their time.

### Ethical Approval and Informed Consent

The study was approved by the Institutional Review Board (approval number: 2017/6697) at a major medical center in northern Europe. Informed consent was obtained after the participants were given information about the nature of the study and aspects of participation.

### Data Collection

Although this study was exploratory and open in nature, the research was conducted with a specific outcome—the development of an eHealth intervention. The interview and analyses were therefore guided by the aims of the study. Semistructured interview guides (Multimedia Appendix 1) were developed by the research team and used to explore participants’ (patients’ *and* spouses’) everyday life with chronic pain, including everyday routines, challenges, and coping strategies; participants’ experiences and thoughts about technology and health-related technology, including smartwatches, activity and nutrition trackers, mindfulness apps/videos, health forums and blogs, and other health-related apps; and participants’ thoughts and expressed needs related to an eHealth pain management intervention. Participants (ie, patients and spouses) were interviewed individually to capture patients’ and spouses’ views separately. Participants were also asked to complete a brief questionnaire assessing background information, including age, type of diagnosis, and treatments in addition to experiences with technology. A patient representative provided input on the background questionnaire and the interview guides to ensure easily understandable and nonoffensive questions. The interviews were audio recorded and conducted face to face by the first author (ILS) and conducted either at the research center, in meeting rooms at local patient education centers, in participants’ home, or at participants’ workplaces. The first two interviews were conducted by the first author together with an experienced interviewer (CV).

### Data Analysis

Interview data were transcribed verbatim and analyzed using a thematic analysis approach in a stepwise process [33]. In the first step, data were deductively grouped into broad themes derived from CBT themes (eg, health-promoting behaviors, thoughts and feelings, and social relations). Second, sentences and longer semantic units were coded and grouped into categories across and within the initial themes. This was done on a manifest as well as a latent level, so that what the respondents actually said as well as the underlying assumptions were analyzed [33]. Each category was then examined again, with a focus on identifying variations, similarities, and differences within each category. Subcategories (ie, minor themes) were identified and named by characterizing content. Finally, all subcategories were examined again and grouped into main categories (ie, major themes). To ensure trustworthiness, the research team consisting of the first author (ILS) and coauthors (LSN, CV, OK, and HE) met regularly to discuss and refine the analysis until an agreement was reached [34]. NVivo 11 (QSR International, Victoria, Australia) qualitative analysis software was used to organize and facilitate the analysis. To increase the transparency of the interpretation, categories and subcategories were illustrated with quotations.

## Results

### Overview

A total of 20 persons (15 women) living with different types of pain and 5 spouses (2 women) were included in the study. The participants’ (patients’ *and* spouses’) age ranged from 18-74 years, with a median of 48 years. Many patients noted that it took some years before they received a diagnosis, with some having lived with pain for 10 years or more before reportedly getting their diagnosis. Almost all patients had been through a variety of treatments, ranging from primary care (eg, general practitioners’ visits and physical therapy) to more specialized treatments and rehabilitation in secondary and tertiary care. There were no clear differences in the results between male and female participants in this study, apart from women describing their pain by using more metaphors. Table 1 presents a detailed view of the patient demographics.

**Table 1:** Patient demographics (N=20).

Characteristic		Number of patients (%)
**Employment status**		
	Working/studying full-time	5 (25)
	Working/studying part-time	4 (20)
	Currently on sick leave	5 (25)
	On disability benefits	6 (30)
**Type of pain**		
	Neck and back pain	8 (40)
	Neurological pain	8 (40)
	Others	4 (20)
**Reported time living with pain (years)**		
	0-3	2 (10)
	4-8	5 (25)
	9-15	5 (25)
	16-25	4 (20)
	≥26 years	4 (20)

**Figure 1:**
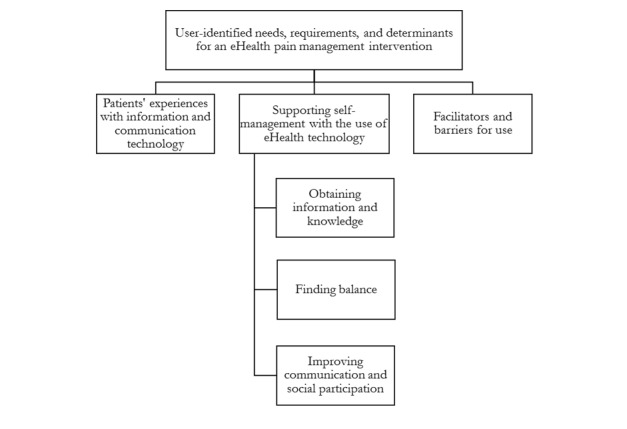
Overview of the main categories and subcategories from the analysis. ICT: information and communication technology.

The findings provided (1) insight on patients’ experiences with ICT, (2) understanding of how an eHealth intervention could support patients’ everyday needs and challenges (ie, support self-management), and (3) information on the facilitators and barriers for patients’ use of an eHealth pain management intervention. Categories 1 and 3 were analyzed on a manifest (visual) level, while category 2 was analyzed on a latent (interpretative) level. Main categories and subcategories are illustrated in Figure 1.

### Patients’ Experiences with Information and Communication Technology

All participants reported owning a smartphone, computer, or tablet and stated that they were using apps regularly, mostly for practical day-to-day use such as buying a bus ticket or for checking the weather forecast. Nearly everyone had previously downloaded an app, and about half (n=11) reported having used a health-related app, mainly mindfulness apps for relaxation and focus control, or apps for step counting. Those who had tried apps for step counting often stopped using them, as they felt as if they were never able to walk enough because of their pain. Some, especially those under 35 years of age, also regularly used apps for podcasts as a strategy to distract focus away from their pain. Only two participants had tried apps specifically for pain management, mainly with a focus on migraine, and rarely, apps were offered in the participants’ native language. Many stated that they did not find or know of any apps specifically for pain management. A few participants had used YouTube for videos showing mindfulness, breathing, and visualization exercises. Mindfulness exercises (using apps, YouTube, or CDs) were particularly used at night when in bed as a relaxation strategy when trying to sleep. Participants who had not tried any health-related programs (eg, apps, smart watches, or similar) reported not having thought about it, not finding any apps relevant enough for their needs, or not knowing what to download and use.

The majority of the participants described using their mobile phone more than their tablets and computers. They preferred to use their phone for apps, as they found that apps were more easily available on the phone. Tablets were used when searching for information or reading news, while computers were mostly used at work or for practical tasks such as paying bills. Some also reported using their computer for watching television series, a strategy often used for distraction from pain. Searching for information about pain and treatments on the internet was common, especially during the first years of living with chronic pain, but many emphasized challenges related to finding information on the internet, as they did not know what information to trust, and expressed concern about receiving incorrect or even damaging information or advice. Most participants had checked out different pain forums, blogs, or groups on the internet, but few reported using or participating in these actively; instead, the patients perceiving them as “too negative” and “depressing” to follow *.* Some (n=5) found these types of forums useful when needing advice from peers, emphasizing that they avoided looking at these forums on days with a lot of pain due to the “negative vibes” from these forums. Only three participants reported communicating with their health care providers electronically (ie, through email or patient/physician web pages).

Participants were generally in favor of using an eHealth self-management intervention for chronic pain: Only one patient did not visualize how or when such an intervention could be of use. Participants were enthusiastic about potentially getting an easily accessible tool in their daily lives that could be used for support and distraction from pain, at any time or in any situation. Thus, a mobile app was identified by the participants early on as the preferred platform for use.

### Supporting Self-Management With the Use of eHealth Technology

#### Overview

Analyses showed that participants expressed the need for self-management support. Independent of the type of pain, participants (both patients and spouses) described common challenges in their daily lives including physical, psychological, and social challenges such as fatigue, isolation, depressive thoughts and anxiety, sorrow and guilt, and memory and concentration problems. The expressed needs, including those regarding an eHealth intervention, appeared related to years lived with chronic pain and their “good or bad days,” which depended on daily pain intensity and level of fatigue.

Taking responsibility and experiencing independence were viewed by participants as essential for self-worth, but challenging without the necessary information, knowledge, and support. The informants’ expressions for self-management support through the use of eHealth technology were related to three different aspects: obtaining information and knowledge, finding balance, and receiving support for improving communication and social participation.

#### Obtaining Information and Knowledge

Most participants (ie, patients and spouses) were positive toward, or expressed a wish for, receiving information and gaining knowledge through technology and educational texts, communication with health care professionals, or both. Topics identified included the need for information about pain, in general, and pain physiology; health-promoting behaviors including information about sleep, activity pacing, physical activity, and nutrition; treatment options, medication, and medical aids; psychosocial information including information about communicating pain with others and coping with anxiety and depressive thoughts; and reports on the newest pain-related research.

Many of the participants reported that a lack information about pain and pain management, especially during their first years living with pain, induced a feeling of desperation and anxiety. For instance, many patients had worried about their memory issues, with catastrophizing thoughts of having dementia. As a result, many stated that they had been “shopping” for health care services and alternative treatments, trying “everything” they came across instead of focusing on pain management strategies for themselves. Over the years, many had found their own ways of managing their pain, stating that they did not have the same need for information now as compared to early in the disease trajectory. These patients further emphasized that they wished they had received this kind of information earlier, instead of trying to work out all these things on their own. Compared to the patients who had not lived with pain for very long, these patients wanted more information on the newest research, knowledge, coping strategies, and exercises that could support positive psychological well-being.

Many participants expressed a feeling that health care providers, in meetings with them, lacked knowledge about their pain or gave conflicting information about what to do with the pain. Several participants were told by their physician that chronic pain is difficult to deal with and some were instructed to look up chronic pain on the internet themselves, resulting in further contradictory and sometimes scary information. Two participants had initiated additional education for themselves (ie, studying psychotherapy and medicine) in an attempt to gain an in-depth understanding of their pain and how to best cope with it.

The participants also emphasized the need for independence. Several preferred to work things out by themselves, if possible, yet needed more knowledge to do so successfully. Getting trustworthy information from health care providers and researchers in a self-management app was seen as a positive option that could potentially provide the needed knowledge. Some wanted direct contact with, and support from, health care providers through the intervention, but expressed doubt about how this would work, considering the need for contact with specialized health care providers who were already extremely busy and often unavailable. Participants also described the daytime work hours of health care personnel as challenging, as patients often need help at different hours of the day, not just during daytime hours. However, both patients and spouses considered the potential of having access to information and knowledge through an eHealth intervention as beneficial. The spouses pointed to personal challenges in understanding the pain and suffering caused by pain. They wanted more information about the pain and how it could connect to their partner’s psychological health and well-being, emphasizing the need for knowledge about how to better communicate with each other despite strong emotions or pain. They also described feelings of being left out and helplessness seeing their partner in pain and not being able to adequately help.

The potential for an eHealth intervention to provide information on the patient’s own terms was further emphasized, as the ability to decide when and how much information to receive was considered focal. Table 2 provides a summary of participants’ expressed needs and suggestions for content and functionality, and Multimedia Appendix 2 provides additional quotes from the participants.

#### Finding Balance

A topic addressed by all participants (ie, patients and spouses) was the need for, and the challenges around, finding a balance in everyday life. This particularly included challenges with finding a balance between seeking help versus being independent, being active while getting enough rest (ie, activity pacing), and focusing on and talking about the pain versus distraction from the pain. The majority of the participants had accepted their pain and did not think that it would ever subside, although they found it difficult to cope with and accept the *consequences and impact* of pain on their daily lives. They described challenges around fully understanding how pain is affected by other variables in life, such as sleep, mood, and activity. Despite being familiar with the concept of activity pacing, few participants actually practiced such pacing, stating a lack of competence and knowledge of how to appropriately pace activities. Those who mastered activity pacing had achieved this mastery through repeatedly trying and failing over many years of living with pain, and they wished they had learned about the importance of activity pacing early on. The patients also described challenges about making others understand their pain. They wanted support and advice in this process, so that they could become more balanced and better cope on a day-to-day basis in their pursuit of regaining a “normal” or “new normal” life.

It’s important to feel normal and it’s important to have a NORMAL everyday life. The daily life from before is not yours anymore. You need to find a NEW normal. And in that process, it is important to have a tool, important to have...overview.

patient

Some of the patients had positive experiences with relaxation exercises, stating that they were helping them feel “more balanced.” They wanted such exercises made more available in everyday life, for instance, through a mobile app, specifying that reminders would be helpful, as they often forgot to do such exercises. Others wanted help with focusing on the positive things in life, suggesting that some forms of “words of wisdom” might be useful.

One topic addressed by many was the possibility of doing daily registrations. The patients wanted to be able to register details such as mood, sleep, pain, and activity in order to gain better control over their own coping strategies and skills, rather than always relying on health care services. Providing patients with visual information, for instance, in a graph consisting of their registrations, could give them concrete information that they could learn from, but also share with their partner or health care provider, making it easier for those around them to understand how the pain affects them.

Some of the patients, and especially the spouses, also emphasized that such registrations could be a way of putting things into perspective, as registrations could show them that a variable such as sleep or a situation such as a social event, might not have the impact they thought it would have. One spouse said:

During especially bad times, one feels that this is worse than ever before. And that this will never be better or OK again. And if you then had some clear statistics, something objective. Something that could say; “you were actually doing worse this time last year. But just a month later, you climbed that mountain”.

spouse

Some spouses emphasized that their partner did more than they thought they did, often pushing themselves too hard, sometimes even harder than people without health issues. Registrations could help patients be more satisfied with a lower or a more balanced activity level, suggesting that registrations could support acceptance in daily life. At the same time, some participants also recognized that such registrations could have a negative impact on their mood and self-efficacy (eg, not experiencing “good days” and hence only registering “bad days”).

**Table 2:** Participants’ (N=25) needs, suggestions, and reasoning for content and functionality of eHealth interventions.

Needs	Suggestions for content/functionality	Reasoning
Information and knowledge	Information about pain physiology, treatments and medicine, and legal rights and pain management	Feel safe Feel less uncertainty and desperation Be more independent Help accept the consequences of the pain
	Direct contact with health care providers	Get to ask questions when needed Feel safer Get constructive feedback for better coping and motivation
Balance	Daily registrations of variables like sleep, mood activity, and pain	See how the variables connect and learn from mistakes Get a broader perspective Something to show health care providers/partners for better support
	Pain diary/notes	Save important messages, advice, and experiences from doctor’s appointments, courses, etc Get a broader perspective on personal thoughts and experiences and get help accepting the consequences of the pain
	Calendar	Keep track of appointments regarding pain treatments, to avoid your personal calendar getting too pain focused
	Medical diary/list	Keep track of medical history Bring to health care providers
	Word of the day/word of wisdom	A reminder of what’s most important in life Motivation
Communication and social participation	Social forum or inspirational stories from peers	Feel less alone Get support and advice from peers Become more motivated for change
	Writing three positive things/self-praise	Better mood/be more enjoyable to be with A reminder of what’s most important in life Focus on the things one is able to manage
	Advice on communication or direct contact with partner via the app	Be able to talk about pain at home, possibly experiencing more understanding and support
	Breathing, focus, and relaxation exercises	As a break/distraction from the pain To help become more present in daily activities and social gatherings

Some patients had tried such registrations during clinical pain assessments and treatments or in relation to applying for disability benefits, but found it challenging to remember all these variables or carry a notebook all the time. Using the phone for such registrations was therefore considered an easier and more accessible option. Table 2 provides more details on this, and Multimedia Appendix 2 provides additional quotes from the participants.

#### Improving Communication and Social Participation

Patients’ needs for self-management included a need for normalization and independence. This also influenced their social relations, and the majority described challenges with accepting role changes and finding their new place in social settings. Many described pushing themselves in the attempt to be “like they used to be.”

If I’m not given special attention [due to the pain], I’ve actually achieved what I’m dreaming of. Then I have managed to stabilize everything, so that life is normal. But it’s only a dream.

patient

I’m the mom, NOT the sick mother. That’s extremely important to me...I do everything to be able to be and say that.

patient

Many participants (spouses and patients) described that these attempts of trying to be “like they used to be,” often resulted in increased pain and fatigue, with the patients becoming more distant, irritated, and sometimes angry. The spouses found this difficult to deal with and emphasized that their partner could use help with acknowledging the situation, so that they would not push themselves so hard or so that they could open up to the idea of medical aids and supporting tools. The patients, on the other hand, expressed difficulties getting their partner to understand their needs for normality and independence. One patient emphasized that it was “all about how things were said.” She had experienced positive changes at home after her husband had participated in a self-management course for caregivers. She described him as having gone from being frustrated and accusatory toward her whenever she did too much, to clearly expressing his *own wish* to do those things, thereby making it easier for her to let go. This particular participant, as well as others, emphasized the need for openness, even though most of the participants stated that they did not talk much about their pain at home. They stressed upon the need for a conversation starter and for advice on how to make necessary adjustments to facilitate such conversations.

High pain levels, concentration issues, and fatigue were considered to hinder patients’ social participation, as many felt like they were “not present enough” or that they were “too aggressive.” The need for something that could distract and give them a break from their pain was emphasized. A reminder to perform a breathing exercise, perhaps, after work before sitting down for dinner with the family, was one suggestion. Several stated that they did not want health care providers to follow their progress in these tasks, as they felt this could pressurize them in a negative way, since they already feel as if they never did enough.

Some participants also suggested that being able to connect with peers through a type of social forum could be a useful functionality, making them feel less alone and less frustrated. They did, however, state that they did not want any form of “competition” with peers. Several hoped for inspiration from others through social forums and to learn from their experiences, either directly through a forum or as stories to read and choose from. When asked why they imagined using a forum in the eHealth intervention when they did not want to participate in forums/pain groups online, some answered that they thought this could be a more positive experience, as the intervention likely would target those who wanted to learn to cope with the pain, rather than “complain about it.” Table 2 provides more details on this, and Multimedia Appendix 2 provides additional quotes from the participants.

### Facilitators and Barriers for Use

Participants were also asked to reflect upon what might make an app “good or bad” in their opinion and any thoughts they might have about potential facilitators and barriers for use of an eHealth self-management intervention for chronic pain. A summary of topics discussed and emphasized by the participants around this are presented in Table 3 (for additional quotes from the participants, see Multimedia Appendix 2). Participants seemed to agree that “good apps” are those that are user-friendly and easy to use; informative and functional, without being overwhelming (several participants had stopped using apps because they felt they were too overwhelming); and without errors or too many updates. Regarding an eHealth self-management intervention, accessibility was addressed as an important facilitator to allow patients to easily access the coping resources they needed when experiencing elevated pain and fatigue levels. Suggestions included having a simple login procedure, emphasized by many as a necessity for use. Between options of a variety of functionalities (eg, a journal, forum, and social connection) or a simple login, 15-20 patients chose a simple login.

Participants did, however, emphasize the importance of having a variety of options to choose from, related to design features and content units, particularly considering their pain and concentration challenges, making the intervention more personalized. For example, they suggested that the intervention could give them advice about content, topic, and exercises to perform based upon their daily registrations. Alternatively, they wanted to be able to choose the content topic and duration of use, for instance, through the use of a “read more” button and an exercise list. This could also allow them to choose exercises or brief educational texts on “bad days” with high pain and concentration issues. The participants also emphasized the need for reliable and up-to-date information and stated that they had chosen to participate in this study, as they felt safe knowing that health care professionals were involved in the intervention development. Poor usability, including nonintuitive design and an overwhelming amount of information, was emphasized as the main barriers for use.

**Table 3:** Facilitators and barriers for the use of eHealth interventions.

Themes/topics	Facilitators	Possible barriers
Accessibility and privacy	Mobile app for everyday use, tablet for longer reads	Computer and tablet not accessible enough throughout the day
	Simple login; code is acceptable and trustworthy	Cumbersome login procedure
Usability	Simple design with intuitive icons	Complex icons or background noise that could disturb the concentration level
	Easily readable and short texts, preferably with a “read more” button	Longer texts that challenge the concentration level
Personalization and tailoring	Daily registrations (ie, sleep, mood, and pain) for more personalized content based on needs and challenges	Overall lack of personalization
	Reminders: ability to choose when and how	Continuous reminders with “bad timing”
	Voice-overs: possibility of choosing between different voices	A “wrong” voice
Reliability of the intervention	Evidence-based content and involvement of specialized health care professionals	None
	Updates and up-to-date information	Never any new content
	Supportive and nonjudgmental language	Judgmental, negative, or “glossy” language

## Discussion

### Principal Findings

Identifying and exploring patients’ needs, experiences, and preferences are of essence when designing and developing eHealth interventions. In this study, patients with chronic pain and some of their spouses participated in the early stages of developing an eHealth pain management intervention. The participants (ie, patients and spouses) provided insight into patients’ experiences (and from the spouses’ point of view, patients’ perceived experiences) with ICTs, their needs and challenges in relation to an eHealth intervention, and their thoughts about possible facilitators and barriers for the use of an eHealth pain management intervention.

Participants were generally in favor of using an eHealth self-management intervention for chronic pain and considered such a potential eHealth tool acceptable for gathering knowledge and gaining support related to pain management. Despite previous experience with health-related apps (eg, mindfulness apps and exercising apps) or online pain groups, participants described a lack of existing eHealth apps and interventions specifically targeting chronic pain and pain management. Participants were enthusiastic about the prospect of obtaining a tool targeting pain management and receiving information as well as useful exercises, and through them, “getting everything you need in one place.” Easy access and availability were regarded necessities for use, with participants depicting a mobile app as the preferred platform for use.

Regardless of the type of pain, participants described comparable challenges in their daily lives, including fatigue and sleeping challenges; memory and concentration issues; and psychosocial challenges such as negative thoughts, anxiety, guilt, sorrow, and feelings of isolation, all supporting the existing literature [1,2,35]. Participants expressed a need for obtaining more information and knowledge, finding a balance in everyday life, and receiving support for improving communication and social participation. For an intervention to best address and facilitate support for these needs, the participants also provided suggestions for the eHealth intervention content and functionality.

Possible facilitators and barriers for patients’ use of an eHealth intervention were also identified, with participants emphasizing that an accessible tool could be used in any circumstance, regardless of the varying pain level or concentration issues, as a likely facilitator for use. Poor usability and limited or lacking tailoring or personalization were identified as the potential main barriers for use.

### Providing Self-Management Support Through eHealth

Acceptance is considered a necessary component when teaching self-management skills, as individuals who have accepted their pain are more open and willing to take an active role in the self-management process [7]. The majority of the patients in this study described having accepted their pain, suggesting that they were open for self-management education and support. This was also reflected in their interest in eHealth interventions for self-management and their expressed need for self-management strategies. The participants (ie, patients and spouses) expressed needs that reflect common content and themes from existing face-to-face CBT interventions targeting chronic pain, including a wish for more knowledge about recommended health-related behaviors, activity pacing, and psychosocial support. Patients also emphasized a need for independence and normalization, yet expressed a wish for support, or as one participant said, “some tools” that could be helpful in this process. This supports the notion that patients prefer self-management support [4] in order to respond to physical and mental changes and to manage their day-to-day challenges and decisions.

Patient empowerment involves a process to enable patients to have more influence over their health by promoting their capacities to gain control over self-defined important matters, thus leading to better self-management [36]. As such, supporting patients in the self-management process also includes helping patients become more knowledgeable and at the same time, assisting them in feeling empowered (ie, more confident in their skills to manage the illness) [24,37,38]. The participants in this study emphasized that obtaining knowledge and learning management skills through an eHealth intervention could be an accessible way of gaining knowledge, providing them with a constant option they could use at their own preferred time and pace, allowing for self-directed repeated exposure to the information and making the process familiar. Providing knowledge and support through the use of ICT could give patients the opportunity to reinforce knowledge and skills over time, possibly prolonging positive effects over time. In that sense, eHealth interventions could strengthen potentially fading effects of CBT on pain and function [9,39]. Existing research has supported this notion and found internet-based interventions to be a viable way for patients to obtain skills and knowledge, even after formal treatment is completed [12], showing long-term effects [16].

Providing information alone, however, is not sufficient if the goal is to provide pain management and behavioral change [24]. The participants in this study supported this notion, describing a need for a variety of content and functionality in order to become better at managing their own pain.

### Self-Monitoring to Support Balance and Activity Pacing in Everyday Life

Balancing everyday life and pacing activities were some of the main topics emphasized. Although they were familiar with the concept of activity pacing, few participants actually practiced this activity. People with chronic pain sometimes avoid physical activity due to a fear of increased pain, but typically also push themselves too hard despite the experienced pain. Either approach will likely result in poorer overall functioning across time [7]. To enhance awareness of how activities and mood might be related to their pain, and support actual behavior change, the participants in this study suggested establishing daily registrations of variables such as sleep, mood, and activity. Keeping a medical diary for tracking one’s own medical history and writing a daily pain journal were other suggestions. These suggestions are all related to awareness and potential for identifying patterns, helping patients become more aware of their own actions and behavior, which is in line with self-management and CBT goals as well as fostering of health behavior change. Electronic registrations and notes have also gained acceptance as methods for supporting patients in keeping a more reliable and up-to-date diary, instead of one based on recollection [40,41]. These findings are consistent with research identifying factors that determine the success and failure of eHealth interventions, indicating self-management and empowerment as the most important factors for successful outcomes [42]. Studies have also supported the notion of technology-based interventions leading to patient empowerment, as they can encourage patients to take more ownership over their personal health [43] and reduce patients’ dependency on health care services [38].

### Design Features to Support Motivation and Usage

Motivation to complete and continue the use of an eHealth intervention is crucial in order to obtain an effect. Participants in this study expressed interest in eHealth interventions for chronic pain, but despite such interest [19], previous studies reported high attrition and dropout rates for eHealth pain management interventions [44,45]. There is still a need for more information related to acceptance or intended use of eHealth interventions and to adherence [46]. In a study featuring an unguided eHealth pain management intervention, high acceptance did not result in high uptake or adherence [47]. Guided eHealth interventions appear to have less attrition and drop-out challenges [48]. Having a therapist following the treatment could be motivating, allowing for more personalized treatment and important feedback [48]. However, a recent review found only a small difference between self-guided and therapist-guided interventions in relation to drop-outs [15]. The participants in this study had different opinions on the value of or need for a therapist’s support. Some of the participants expressed a wish for contact with health care providers throughout the intervention, particularly in case of arising questions. At the same time, several participants stated that they did not want health care professionals to follow their progression on tasks or exercises, as this could increase the pressure on them, enforcing their frequent and collective feeling of “never doing enough.” All participants emphasized that they wanted the intervention to give them positive input and support, noting that they did not want an intervention that focused on their problems or reminded them of all the things they were not able to do. This supports the notion that close attention should be given to designing a positive user experience with persuasive and engaging features to trigger a positive effect and potentially promote adherence [49].

Examining potential facilitators and barriers for use of an eHealth tool in line with recommendations [42], we found that accessibility, usability, personalization, and reliability were factors that were emphasized by participants as important facilitators for use, supporting the existing literature [21,29]. Poor usability, including a flawed design and an overwhelming amount of information, was emphasized as key barriers for use. The participants emphasized the need for a tool they could use daily, independent of varying pain intensity, activity level, and concentration capacity. These results support previous findings where patients with chronic pain described high demands related to the design of Web-based pain management interventions, even higher than their physicians, indicating challenges for distracting content and texts that were too dense [50].

### Strengths and Limitations

Several aspects of “trustworthiness” [51] were covered well in this study of qualitative research. The credibility was assured by presenting the steps in the analysis as thoroughly as possible as well as showing examples with quotes in the Results section. Transparency was assured by researcher triangulation and by presenting citations from the participants. Dependability was assured by describing the analytical process in detail in the study to make it possible for the reader to agree with and understand the logic of the findings.

This study had several limitations. First, only five spouses were included in this study, and the results might have been different if more spouses had participated. However, the patients were the main focus of the study. Spouses were included to gain additional perspectives on patients’ needs, and adding their voice was seen as a study strength. Second, most of the patients participating were women, and the results may therefore not fully cover the male perspective of how eHealth interventions could support them. Third, most participants had lived with chronic pain for a long time, which may have influenced their description of everyday challenges and needs. Many had also participated in self-management courses, which meant that they already knew a lot about pain and pain management. As such, patients with a shorter history of chronic pain could have other needs and preferences not covered in this study.

Study participation may be due to higher motivation and engagement in self-management than the average patient with chronic pain. Most participants were interested in how they could learn to live and better cope with the pain, rather than how they could get rid of the pain, indicating acceptance and, perhaps, maturity in how this sample approached their pain. Finally, it is not unreasonable to assume that those willing to participate in the study were people with a special interest in technology, as half of the participants had already tried some forms of health-related apps/technology. The results might therefore not fully capture facilitators and barriers that apply to a less technology-experienced group. However, considering the pervasive use of technology in today’s society, most patients with chronic pain likely have some form of ICT experience, and the clear requirement of accessibility and usability should be representative regardless of technology experience. Future studies should explore these topics further, examine how current eHealth interventions can adequately address known challenges with interventions (eg, adherence), and seek to incorporate existing and new findings into the design and development of new eHealth self-management interventions.

### Conclusions

To our knowledge, this is the first study to explore the experiences of patients with chronic pain with regard to ICT, understand how an eHealth intervention can support the everyday needs and challenges of patients with chronic pain, and identify the possible facilitators and barriers for patients’ use of an eHealth pain management intervention. The participants (ie, patients with chronic pain and their spouses) considered ICT an acceptable way of gathering self-management support, particularly emphasizing the need for more information and knowledge, finding a better balance in everyday life, and obtaining support for improved communication and social participation. The participants described an ideal eHealth intervention as one that could be used for self-management support and distraction from pain, at any time or in any situation, regardless of the varying pain intensity and concentration capacity. The results provide insight into the future potential of eHealth interventions aiming to support self-management for patients with chronic pain.

## Appendix

Multimedia Appendix 1 Interview guides.

Multimedia Appendix 2 Additional quotes from participants (related to Tables 2 and 3).

## Abbreviations

CBT: cognitive behavioral therapy
eHealth: electronic health
ICT: information and communication technology
