# Supplementary material for: Patients’ Needs and Requirements for eHealth Pain Management Interventions: Qualitative Study
Source: J Med Internet Res. 2019 Apr 1;21(4):e13205. doi: 10.2196/13205 (PMC6462891; doi:10.2196/13205)
Supplement: Multimedia Appendix 1 [file jmir_v21i4e13205_app1.pdf]

Interview guide: Patients

**Everyday life and coping**	**Daily life**	**A general day: yesterday**	
		Activities and routines	Daily routines: Physical activity, sleep, nutrition
		Good days/bad days	How are they, what contributes to one or the other
		Coping strategies	What are the biggest challenges and how are these met
		Values in life	Most important to you
		Life situation in 6 months	How do you see..
	**Understanding the pain**	Describe the pain	Could you describe your pain?
		Support from family and friends	How would they describe your pain?
		Healthcare professionals/healthcare system	
	**Needs**	Now and earlier	What's been covered well and where
			What's been missing
			Unanswered questions
**Technology**	**Technology use**	Describe your use of different technological platforms	Mobile, computer, tablet
		Technology use in relation to pain	What has been used and why *Internet and search engines, social media and forums, blogs, apps and other e-health solutions
			What makes you continue to use/stop use Three things you like about mentioned solutions
			Communication with healthcare professionals through technology: attitudes and experiences
	**The e-health intervention**	Content	What could it include to be useful to you?
		When and where	
		Design	
		Personal security and privacy protection	

	**Ending the interview**	What do you think is of essence when starting this development process?
		Anything you miss?
		Other things on your mind?

For interview guide spouses, please see next page.

Interview guide: Spouses

**Everyday life and coping**	**Daily life**	Daily life as a caregiver	Describe<br>Habits, changes
		Good days/bad days	How are they, what contributes to
		Challenges, strengths and coping strategies	Biggest challenges (areas *pain symptom circle)<br>How have challenges changed over time
		Values in life	Most important to you
		Life situation in 6 months	
	**Understanding the pain**	Describe partner's pain	
			How does your partner understand his/her pain?
		Communication at home	Describe communication at home
			Do you talk about the pain at home?
	**Needs**	Your needs	What's been covered well and where
			What's been missing
		Your partner's needs	Unanswered questions
**Technology**	**The e-health intervention**	The self-management tool platforms	What could it include to be useful for your partner?
			What would you like in a tool like this?
		Technology usage in relation to health	User experiences – why/why not?
			What makes you continue to use/stop
		Usage: When and where	Think about your partners pain and daily life, are there any situations where this could be relevant? When?
		Design	
	**Ending the interview**		What do you think is of essence when starting this development process?
			Anything you miss?
			Other things on your mind?
