# Supplementary material for: Patients’ Needs and Requirements for eHealth Pain Management Interventions: Qualitative Study
Source: J Med Internet Res. 2019 Apr 1;21(4):e13205. doi: 10.2196/13205 (PMC6462891; doi:10.2196/13205)
Supplement: Multimedia Appendix 2 [file jmir_v21i4e13205_app2.pdf]

Additional quotes from participants (related to Table 2 & 3)

Participants' needs, suggestions and reasoning for content and functionality

Information & knowledge

«I'm very interested in learning about my pain (…) There are some things I have learned… in cooperation with a therapist (…) in relation to the body's nerve system and things like that. And after that, I got an epiphany on a couple of things, and it triggered me in some sense in that (…) I understand more about how my body works and things aren't as scary as they might seem."

Contact with healthcare providers:

«There are naturally a number of questions you are dealing with in relation to pain treatments and what to do or why I react the way I do. One is left with a lot of questions! So, the opportunity to ask questions is certainly present and desirable."

Balance

Daily registrations, notes and/or calendar:

"For me, it's about quickly finding out whether I am in a bad pattern now, have I taken on too many tasks and done too much (…) And then I need to have the possibility to find out, shucks, this has worked well. What's going on now."

Notes:

«I think that it *(writing)* could actually be good for periods of time. Like when I was depressed, it was okay to write things down and get it over with. I do have a tendency to go around and around in my own thoughts, and in some way never finish that thought."

Medical diary/list:

«What I have perceived as important would be to have a database on the types of medicine one has used. The medicines one uses. How much. And how well they work. Over the years it really becomes a lot." - *spouse*

Word of the day/word of wisdom:

"There could be like… call it today's quote. Today's word of wisdom. Not wisdom… not like fortune telling. More like today's awakening. Or this weeks' awakening. A small thing that explains, reminds you to… breathe…"

Communication and social participation

Social forum or inspirational stories from peers:

«I think a chat-room function would be the most important for me. Because… yes, it's like I'm saying, the possibility of being able to just send in like, "this morning it's going to be rain, is there anyone else who feels bad?". If you need it."

Writing three positive things/self-praise:

«What if you had some kind of word-association. If one could choose to get only positive words, then one could associate further in some way. Like turning one's focus onto something else."

Multimedia Appendix 2: Additional quotes from participants (related to Table 2 & 3)

Advice on communication or direct contact with partner via the app:
«Having something to look at together. That is designed in such a way that one could use it as a foundation for communication. That you're in it together…"

Breathing-, focus- and relaxation exercises:
«Breathing exercises and other exercises like that (…) they help me at least. Very important considering that the pain increases, one gets more tense, ties everything up. Shoulders all the way up to your ears. And then it's nice, it's VERY nice, to have an app to turn to."

Facilitators and barriers and for use

Accessibility and privacy:
«I would like to have it *(intervention program)* on the phone. I bring the phone with me all day."

«What I'm thinking in relation to pain management is that it's perhaps something you often need when your pain is really bad. And I know at least with myself that when my pain is at it's worst, then *I* don't really work. My concentration is bad and I can't focus well and I get easily irritated. So if things become cumbersome in some way, and there are things you need to get through to get where you need. Then I think I would have been frustrated and though ugh, why do I need to do this right now. But if it were easy to get in, and in a way think that now I'm in great pain, I need to go straight in and find that exercise I've worked on in the app."

Usability:
«It has to be easy to use. If things are too difficult then I only get furious or impatient, and then I throw the phone against the wall (…) There should at least never be any problems with login and typing in password after one week and renewing password after a month or so."

Personalization and tailoring:
«I think it would be useful if the app could map out what you need. And for instance, over time also notice what you use the most. So that the app in a way could be personalized and adjusted to best fit me."

Reminders:
«And there could be like a positive reminder: "Have you remembered to rest today", "Have you done that breathing exercise today".

Reliability of the intervention
«Well, it can be as simple as being a part of the public healthcare service. Trust and faith in public services in a way. That there are no one making money on it. Yes. And that there isn't a one-person business behind it, but rather complex expertise. So I know that, as I did with this (project), I looked at the organizational map, checked out which actors were involved, are there any names I've seen before… that there are more, that is, a breadth of competence! That's important. That should be transparent."
